# The role of necroptosis in disease and treatment

**DOI:** 10.1002/mco2.108

**Published:** 2021-12-20

**Authors:** Xiaoxiao Liu, Xin Xie, Yuanyuan Ren, Zhiying Shao, Nie Zhang, Liantao Li, Xin Ding, Longzhen Zhang

**Affiliations:** ^1^ Department of Radiation Oncology Affiliated Hospital of Xuzhou Medical University Xuzhou Medical University Xuzhou Jiangsu Province P. R. China; ^2^ Jiangsu Center for the Collaboration and Innovation of Cancer Biotherapy Cancer Institute Xuzhou Medical University Xuzhou Jiangsu Province P. R. China; ^3^ Cancer Institute Xuzhou Medical University Xuzhou Jiangsu Province P. R. China

**Keywords:** disease model, inhibitor, MLKL, Necroptosis, RIPK1, RIPK3

## Abstract

Necroptosis, a distinctive type of programmed cell death different from apoptosis or necrosis, triggered by a series of death receptors such as tumor necrosis factor receptor 1 (TNFR1), TNFR2, and Fas. In case that apoptosis process is blocked, necroptosis pathway is initiated with the activation of three key downstream mediators which are receptor‐interacting serine/threonine protein kinase 1 (RIPK1), RIPK3, and mixed lineage kinase domain‐like protein (MLKL). The whole process eventually leads to destruction of the cell membrane integrity, swelling of organelles, and severe inflammation. Over the past decade, necroptosis has been found widely involved in life process of human beings and animals. In this review, we attempt to explore the therapeutic prospects of necroptosis regulators by describing its molecular mechanism and the role it played in pathological condition and tissue homeostasis, and to summarize the research and clinical applications of corresponding regulators including small molecule inhibitors, chemicals, Chinese herbal extracts, and biological agents in the treatment of various diseases.

## Graphical abstract

Necroptosis is activated by a unique caspase‐independent signaling pathway and mainly relies on RIPK1/RIPK3/MLKL complex. Over the past decade, necroptosis has been found widely involved in life process of human beings and animals. In this article, we attempt to explore therapeutic prospects of the necroptosis regulators in health maintenance and disease treatment by describing the molecular mechanism of necroptosis and the role it played in disease and tissue homeostasis, summarizing the research and clinical applications of corresponding inhibitors and inducers which include small molecule inhibitors, chemicals, Chinese herbal extracts, and biological agents in the treatment of a variety of diseases including cancer, inflammation, infection, ischemia‐reperfusion injury, neurodegenerative diseases in various organs including cardiovascular, pulmonary, liver, pancreatic, renal, enteric, ocular, joint, and skin.

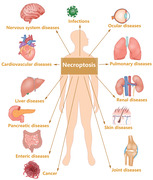

**Abbreviations**
AD: Alzheimer's disease
AEC: airway epithelial cell
AKI: acute kidney injury
ALD: alcoholic liver disease
ALI: acute lung injury
ALS: Amyotrophic lateral sclerosis
AMD: Age‐related macular degeneration
AP: Acute pancreatitis
ARDS: Acute respiratory distress syndrome
ARHL: Age‐related hearing loss
CaMK II: calmodulin‐dependent protein kinase II
cIAP1: cellular inhibitor of apoptosis protein 1
CKD: chronic kidney disease
CMV: Murine cytomegalovirus
CNS: central nervous system
COPD: chronic obstructive pulmonary disease
CS: cigarette smoke
DAI: DNA‐dependent activator of interferon
FADD: Fas‐associated death domain
FasL: Fas ligand
HSV: herpes simplex virus
IPF: Idiopathic pulmonary fibrosis
IRI: Ischemia‐reperfusion injury
KPn: Klebsiella pneumonia
MI: Myocardial infarction
MLKL: mixed lineage kinase domain‐like protein
mPTP: mitochondrial permeability transition pore
MS: Multiple sclerosis
NEC: necrotizing enterocolitis
Nec‐1: Necrostatin‐1
OA: Osteoarthritis
PD: Parkinson's disease
PFT: pore‐forming toxin
RA: Rheumatoid arthritis
RGC: retinal ganglion cells
RHIM: RIPK homeotype interaction motif
RIPK1: Receptor‐interacting serine/threonine protein kinase 1
RIPK3: receptor‐interacting serine/threonine protein kinase 3
RPE: retina pigment epithelial
RSV: Respiratory syncytial virus
*S. aureus*
: *Staphylococcus aureus*
SJS: Stevens‐Johnson syndrome
SPn: Streptococcus pneumonia
TAB 2: TAK1‐binding protein 2
TAK1: transforming growth factor‐β activated kinase 1
TEN: toxic epidermal necrolysis
TNFR: TNF receptor
TNF‐α: tumor necrosis factor‐α
TRADD: TNFR1‐associated death domain
TRIF: TIR domain‐containing adaptor protein
VILI: ventilator‐induced lung injury

## 1 INTRODUCTION

**FIGURE 1:**
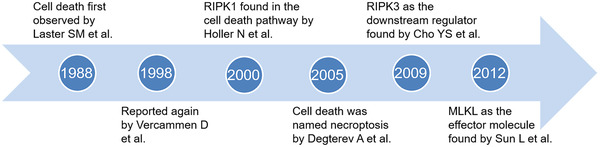
Brief retrospective timeline summary of necroptosis discovery history and milestone events. Necroptosis was first recognized by Laster in 1988. RIPK1 was the first core molecule found in this pathway. The novel cell death was named necroptosis by Degterev in 2005. RIPK3 as the downstream regulator found by Cho. MLKL as the effector molecule found by Sun in 2012

Necroptosis was first recognized in 1988. Laster et al. found that tumor necrosis factor‐α (TNF‐α) could induce a classical form of apoptosis or a necrotic form of cell death.^1^ In 1998, Vercammen et al. found two different Fas receptor‐related pathways: one leads to apoptosis, the other one directs the cells to necrosis when apoptotic is blocked by caspase inhibitors in this pathway.^2^ Subsequently, researchers began to be interested in the molecular mechanism of necroptosis. Receptor‐interacting serine/threonine protein kinase 1 (RIPK1) is the first core molecule found in this pathway. Degterev et al. named this nonapoptotic death pathway induced by Fas ligand (FasL)/TNFR family as necroptosis.^3^, ^4^ Then RIPK3 was proved as downstream regulator,^5^ and the effector mixed lineage kinase domain‐like protein (MLKL) of this pathway was found in a short time^6^ (Figure 1).

As a newly discovered and widely concerned mode of programmed cell death, necroptosis shares the similar morphological characteristics with necrosis such as disintegration of plasma membrane, swelling of organelles, and spillage of cellular contents. Nonetheless, necrosis is a nonregulated programmed death caused by external physical or chemistry stress, such as infection or inflammation at any moment, while necroptosis is a regulated programmed death.^7^ The key molecules in the necroptosis pathway are RIPK1/3 and MLKL.^8^ As a supplementary cell death mode for the failure of cell apoptosis, necroptosis is a characteristic cell death mode activated by a unique caspase‐independent signaling pathway^9^, ^10^ and closely related to homeostasis, inflammation, cancer, neurodegeneration, infectious diseases, cardiovascular diseases, a variety of skin diseases, and acute kidney injury (AKI).^11^, ^12^, ^13^, ^14^, ^15^, ^16^ In this article, we will discuss the function of necroptosis in homeostasis and diverse diseases, and further reveal the application of necroptosis regulators.

### 1.1 The molecular mechanism of necroptosis

Necroptosis occurs when the death receptor tumor necrosis factors (such as TNFR1, TNFR2, and Fas) bind their death receptor ligands (such as TNF‐α, FasL) with inhibition or blocking‐up in apoptotic pathway. The TNF‐α induced necroptosis pathway is generally understood.^17^ Specifically, TNF‐α activates TNFR1 on the cell membrane leading to the structure change of TNFR1. Consequently, TNFR complex is formed by recruiting a series of proteins which includes TNFR1‐associated death domain (TRADD), RIPK1, TNFR‐associated factor 2/5, a cellular inhibitor of apoptosis protein 1(cIAP1), cIAP2, and ubiquitination complex. TNFR complex I then coordinate numerous downstream signaling pathways through complex patterns, such as ubiquitination and phosphorylation, determining the end of the cell (survival or death). The ubiquitination status of TNFR complex I is important for determining many downstream pathways.^18^ Polyubiquitinated RIPK1 recruits transforming growth factor‐β activated kinase 1 (TAK1), TAK1‐binding protein 2 (TAB 2), and TAB3 to form a TAK1‐TAB2‐TAB3 complex which can activate NF‐κB signal pathway for cell survival.^19^ In contrast, the deubiquitination and release of RIPK1 from TNFR complex I promote the formation of TNFR complex II, which can activate downstream cellular apoptosis and necroptosis pathways.^20^, ^21^ TRADD and Fas‐associated death domain (FADD) dissociate from complex I resulting in assembly of complex IIa with pro‐caspase‐8 and RIPK1. Complex IIa promotes the activation of caspase‐8, and activated caspase‐8 causes apoptosis by activating caspase‐3.^22^, ^23^, ^24^ Complex IIb consisting of FADD, RIPK1, and pro‐caspase‐8 is independent of TRADD and could induce caspase‐8‐dependent apoptosis.^25^, ^26^ With caspase‐8 inhibition, RIPK1 binds RIPK3 via RIPK homeotype interaction motif Rhim (RHIM) containing consensus sequences IQIG (RIPK1) and VQVG (RIPK3) to form complex II c/necrosome, the functional amyloid proteins required for signal transduction and activation of necrosis. Furthermore, it plays a critical role in initiating necroptosis.^27^ After the initiation of necroptosis, necrosome promotes the formation of disulfide bond‐dependent MLKL polymer by activating the phosphorylation of pseudokinase MLKL on threonine 357/serine 358 (in humans) or serine 345 (in mice),^6^, ^28^ resulting in the assembly of RIPK1‐RIPK3‐MLKL complex. As a result, MLKL is endowed with the ability to translocate to the plasma membrane to cause cell rupture and the execution of necroptosis.^29^ Other death receptors, such as Fas, mainly recruit membrane‐associated death complex composed of FADD and caspase‐8 after being connecting with its ligand.^30^ In the presence of cIAP and the absence of caspase‐8, Fas also promote the formation of necrosome by a RIPK3‐dependent means. Necroptosis is process to eliminate cells that fails to perform apoptosis. In other words, it can be considered as a supplementary cell death mode of apoptosis.^31^

The regulation of the necroptosis signaling pathway mainly targets at three key molecules, RIPK1, RIPK3, and MLKL. The unique protein‐protein interaction domain on the C‐terminus of RIPK1 and RIPK3 is known as RHIM, mediating the interaction between RIPK1 and RIPK3.^27^, ^32^ Although RIPK1 is a signal molecule necessary to activate RIPK3 in the TNF‐induced necroptosis pathway, it is not a prerequisite for other necroptosis pathways. RIPK3 can open the mitochondrial permeability transition pore (mPTP) by activating Ca2^+^/calmodulin‐dependent protein kinase II (CaMK II). The mPTP opener eventually leads to necroptosis of cardiomyocytes.^33^ In addition to being activated by RIPK1, RIPK3 can also be activated by other proteins containing RHIM domain including TIR domain‐containing adaptor protein (TRIF) and DNA‐dependent activator of interferon regulatory factors (DAI).^34^ For instance, toll‐like receptor 3 (TLR3) or TLR4 directly activates TRIF‐RIPK3‐MLKL pathway of necroptosis through the synergistic action between TRIF containing RHIM and RIPK3.^35^ RIPK3 is a signal integration molecule that can be used for necroptosis demand. It can interact with other RHIM‐containing signal molecules through RHIM, leading to different necroptosis pathways.^36^, ^37^

**FIGURE 2:**
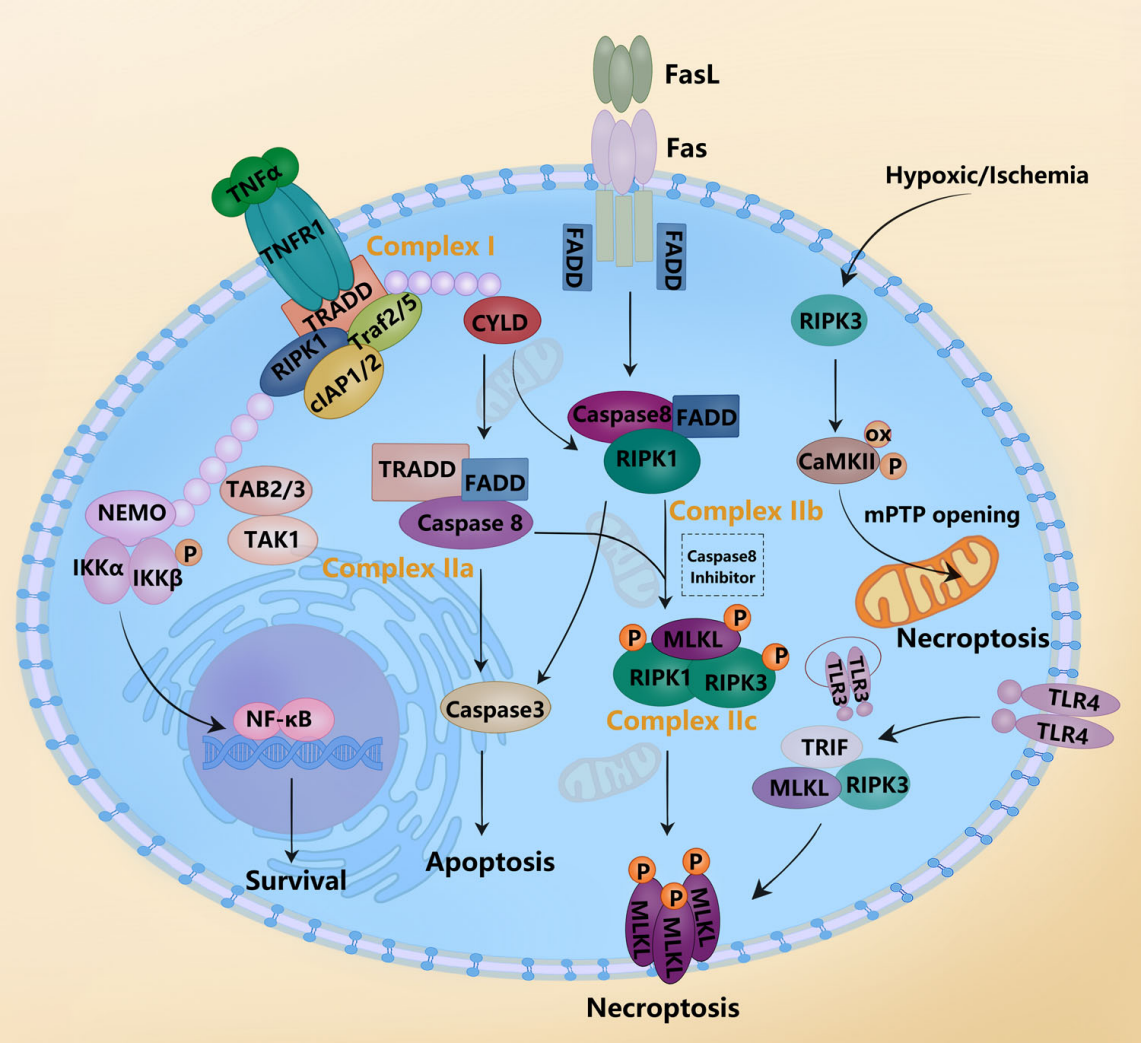
Signaling pathway of cell survival, apoptosis, and necroptosis. Necroptosis occurs when the death receptor tumor necrosis factors such as TNFR1, TNFR2, and FAS bind their own death receptor ligand such as TNF‐α, FasL with a inhibition or blocking‐up in apoptotic pathway. The deubiquitination and release of RIPK1 from TNFR complex I promotes the formation of complex II, which can activate downstream cell apoptosis and necroptosis pathways. To induce necroptosis, RIPK3 can trigger the mPTP by activating CaMK II

MLKL is considered to be a pseudokinase based on the lack of two of the three conserved catalytic residues on its kinase‐like domain.^38^ MLKL activation is a continuous process starting with kinase‐like domain dimerization followed by the internal coiled‐coil region self‐assembly and final formation of appropriate MLKL oligomer. The direct outcome of RIPK3‐induced MLKL phosphorylation is the dimerization of the kinase‐like domain of MLKL. The necessary condition for MLKL oligomerization and function guarantee is that MLKL self‐assembles through the internal coiled‐coil region. Besides human MLKL, functional and structural analysis showed that kinase‐like domain dimerization is conserved in mammalian species, indicating a common step in the process of RIPK3‐induced MLKL activation.^39^ With the ability to directly bind to lipids, polymerized MLKL compromise the integrity of cell membrane by forming membrane permeability pores, which results in necroptosis.^17^, ^40^ Besides necroptosis, recent researches show that MLKL also cross‐talks with other regulatory cell death pathways, translocates to the nucleus to regulate gene expression, binds lipids to destroy bacteria, and inhibits specific metabolic processes and tissue regeneration.^41^, ^42^ (Figure 2).

### 1.2 The characteristic difference between necroptosis and apoptosis

Both necroptosis and apoptosis are regulated by the machinery at molecular level. Apoptosis is a normal biological phenomenon that most cells in the body can keep a certain developmental procedure to maintain a stable internal environment. While apoptosis is a highly regulatory process of insoluble cell death induced by caspase family, necroptosis is activated by a unique caspase‐independent signaling pathway mainly depending on RIPK1/RIPK3/MLKL complex. The morphological features of apoptosis include cell shrinkage, cell membrane blebbing, chromatin condensation, apoptotic body formation, and rapid engulfment by surrounding phagocytes. No overflow of contents occurs during apoptosis, hence, no inflammatory immune response. In contrast, necroptosis is a kind of cytolytic death. Due to the rapid loss of the plasma membrane integrity, proinflammatory content leak out of the cells causing a wide range of inflammatory reactions^43^, ^44^ (Figure 2).

### 1.3 Necroptosis in homeostasis and diseases

Necroptosis regulation is crucial for maintaining tissue homeostasis and preventing inflammation.^45^ The loss of key proteins in the apoptosis pathway, such as caspase‐3, apaf‐1, caspase‐7, and Bax/Bak, has minimal effect on embryonic development. The deficiency of caspase‐8 and FADD, however, is fatal to embryonic cells at around E10.5 embryonic day. The explanation of this phenomenon has remained controversial until researchers discovered that caspase‐8 and FADD are needed to suppress RIPK1 and RIPK3 expression during early embryonic development. Ablation of RIPK3 completely rescued the fateful death of caspase‐8‐deficient mice. In line with this, the loss of RIPK1 allowed normal embryogenesis in FADD‐deficient mice. In immune system, necroptosis inhibition by the caspase‐8 and FADD supports the survival of T cells during clonal expansion.^7^ Signaling pathways that control the immune response, cell death, and cell survival have a crucial role in regulating tissue homeostasis and inflammation. FADD and caspase‐8 prevent intestinal inflammation by inhibiting necroptosis. FADD deficiency leads to TNFR1‐mediated RIPK3‐dependent necroptosis contributing to the intestinal barrier destruction and bacterial‐driven inflammation. Necroptosis of epithelial cells may also cause colitis. Inhibition of RIPK3‐MLKL‐dependent necroptosis and FADD‐caspase‐8‐dependent cell apoptosis totally prevents the development of animal skin lesions. Inhibition of the key molecules in necroptosis pathway helps to prevent periodontal disease.^46^ All of the previous researches have shown that inhibiting necroptosis is of importance to tissue homeostasis maintenance and inflammation prevention.^45^

Necroptosis has two sides in cancer and its development. On the one hand, the downregulation of RIPK3 or MLKL is related to poor prognosis of various types of cancer, such as breast cancer, colorectal cancer, acute myeloid leukemia, head and neck squamous cell carcinoma, melanoma, cervical squamous cell carcinoma, gastric cancer, and ovarian cancer. SN38 (topoisomerase inhibitor) is proved to inhibit cell proliferation in colon cancer by inducing DNA damages and promoting necroptosis. On the other hand, the upregulation of RIPK3 or RIPK1 is related to the development of glioma, lung cancer, and pancreatic cancer. Necroptosis can also promote metastasis by promoting inflammation or tumor cells‐induced necroptosis in endothelial cells.^47^ Necrostatin‐1 (Nec‐1), a specific inhibitor of necroptosis, can reduce inflammation and colitis‐related tumor formation^48^ (Tables 1, 2).

**TABLE 1:** The role of necroptosis in human diseases

Organ/System	Diseases	Observations	References
Nerve system diseases	PD	RIPK1, RIPK3, and MLKL were upregulated in the substantia nigra of PD‐derived postmortem tissue	^56^
	AD	RIPK1, MLKL, and pMLKL were increased in human AD brains	^180^
	MS	RIPK1, RIPK3, and MLKL were increased in cortical lesions in human MS brain samples	^60^
Cardiovascular diseases	Aortic aneurysms	RIPK1 and RIPK3 were increased in human abdominal aortic aneurysm	^74^
	Atherosclerosis	The expressions of RIPK3 and MLKL were elevated in humans with unstable carotid atherosclerosis	^77^
Pulmonary diseases	COPD	The pRIPK3, MLKL, and pMLKL were increased in lung of patients with COPD	^51^
	IPF	RIPK3 and p‐MLKL were increased in the lungs of IPF patients	^91^
Liver diseases	Alcoholic hepatitis	RIPK3 was increased in liver tissues of human with alcoholic liver disease	^50^
Enteric diseases	NEC	RIPK1, RIPK3, and MLKL were significantly upregulated in NEC patients tissue	^54^
Joint diseases	Osteoarthritis	RIPK1 and RIPK3 were significantly upregulated in cartilage from OA patients	^121^, ^122^
Cancers	Glioblastoma	The worse prognosis of glioblastoma was associated with upregulated RIPK1	^127^
	Lung cancer	The worse prognosis of lung cancer was associated with upregulated RIPK1	^129^
	MDS	The worse prognosis of MDS was associated with upregulated RIPK1	^128^
	Breast cancer	The better prognosis of breast cancer was associated with increased RIPK1	^131^
	Colorectal cancer	The better prognosis of colorectal cancer was associated with increased RIPK3 and MLKL	^132^, ^133^, ^134^
	Melanoma	The better prognosis of melanoma was associated with increased RIPK3	^135^
	Gastric cancer	The better prognosis of gastric cancer was associated with increased MLKL	^136^
	Ovarian cancer	The better prognosis of ovarian cancer was associated with increased MLKL	^137^
	HNSCC	The worse prognosis was associated with decreased RIPK1	^138^
	CSCC	The worse prognosis was associated with decreased MLKL	^139^
Infections	S. Pneumonia infections	RIPK3‐initiated necroptosis was essential for host defense against *S. pneumonia*	^169^
Skin diseases	SJS/TEN	RIPK3 functioned as a diagnostic and severity marker for SJS/TEN	^179^
	Psoriasis	RIPK1 and MLKL were increased in human psoriasis	^178^

AD, Alzheimer's disease; COPD, chronic obstructive pulmonary disease; CSCC, cervical squamous cell carcimoma; HNSCC, Head and neck squamous cell carcinoma; IPF, Idiopathic pulmonary fibrosis; MDS, Myelodysplastic syndromes; MS, Multiple sclerosis; NEC, necrotizing enterocolitis; PD, Parkinson's disease; SJS, Stevens–Johnson syndrome; TEN, toxic epidermal necrolysis.

**TABLE 2:** The role of necroptosis in animal models of diseases

Organ/System	Diseases	Modeling method	Gene knockout /Inhibitor	Observations	References
Nerve system diseases	Ischemic stroke	Middle cerebral artery occlusion	RIPK1^Δ/Δ^, RIPK1^K45A/K45A^, RIPK3^−/−^, MLKL^−/−^, Nec‐1	RIPK1 kinase‐dead mutants (Nec‐1 inhibitor), RIPK3 deficiency, and MLKL deficiency could protect mice from acute ischemic stroke by blocking necroptosis.	^56^, ^180^
	PD	6‐OHDA injected in the right striatum	RIPK3^−/−^, MLKL^−/−^ Nec‐1	Inhibition of RIPK1 and genetic ablation of MLKL and RIPK3 could decrease dopaminergic neuron degeneration in preclinical models of PD.	^60^
	AD	AlCl3‐induced	Nec‐1, NSA	Nec‐1 and NSA could suppress necroptosis in AD brain.	^62^, ^63^
	ALS	Optn‐/‐mice model	RIPK1^D138N/D138N^, RIPK3^−/−^, Nec‐1	RIPK1 kinase‐dead mutants (Nec‐1 inhibitor), RIPK3 deficiency improved motor performance.	^65^
	MS	Cuprizone	Nec‐1	The demyelination and disease development were inhibited by Nec‐1 in multiple sclerosis.	^68^
	ARHL	Aged mice	None	The protein expressions of RIPK1, RIPK3, and MLKL were increased in aged mice.	^69^
Cardiovascular diseases	Myocardial infarction	LADCA ligation	RIPK3^−/−^ Nec‐1	RIPK1 inhibitor and RIPK3 deficiency could reduce the infarct size.	
	Aortic aneurysms	Elastase	RIPK3^−/−^ Nec‐1, GSK074	Nec‐1 and GSK074, the necroptosis inhibitors, could attenuate the aortic expansion in mouse model.	^74^, ^75^, ^76^
	Atherosclerosis	Apoe (‐/‐) mice model	Nec‐1	Nec‐1 could reduce the degree of in atherosclerosis mice model.	^77^
	Diabetic Cardiomyopathy	Streptozotocin	RIPK3^−/−^	The deficiency of RIPK3 could relieve myocardial injury and improve cardiac function.	^78^
Pulmonary diseases	COPD	Cigarette Smoke	RIPK3^−/−^, MLKL^−/−^, Nec‐1	RIPK1 inhibitor, RIPK3^−/−^ or MLKL^−/−^ mice could attenuate CS‐induced airway inflammation.	^51^, ^81^
	ARDS	Oleic acid	RIPK3^−/−^ Nec‐1	RIPK1, RIPK3, and MLKL were remarkably upregulated in lung tissue of ARDS rat model. Nec‐1 or Knocking out RIPK3 ameliorated the lung tissue injury.	^82^, ^83^, ^84^
	Asthma	Inoculated with RSV	RIPK1^−/−^	Inhibition of the RIPK1 and MLKL could protect the mice from asthma.	^90^
	IPF	Bleomycin	RIPK3^−/−^ Nec‐1	Nec‐1 and RIPK3 deficiency could protect the AECs from Bleomycin by decreasing p‐MLKL expression.	^91^
	Acute lung injury	Ventilator Hyperoxiad	RIPK3^−/−^	Ventilator or hyperoxia‐induced lung injury could alleviate in RIPK3‐deficient model.	^92^, ^93^
Liver diseases	Alcoholic hepatitis	Ethanol	RIPK3^−/−^	RIPK3 deficiency had a protective effect on ethanol liver injury.	^50^
	Acute liver injury	Acetaminophen	RIPK3^−/−^ Nec‐1	Using Nec‐1 or RIPK3 deficient mice could protect the liver.	^102^, ^103^
		Listeria monocytogenes	RIPK1^−/−^	RIPK1 deletion could protect the liver.	^104^
		Concanavalin A	MLKL^−/−^ Nec‐1	Treating with Nec‐1 or MLKL deficient mice could protect the liver.	^105^
Pancreatic Diseases	Acute pancreatitis	Caerulein	RIPK3^−/−^, MLKL^−/−^	The inhibition of necroptosis mediated by RIPK3 and MLKL had a protective effect in AP.	^111^
Enteric diseases	Intestinal injury	Mimic heat stroke	Nec‐1, GSK872	Nec‐1 or GSK872 could significantly reversed intestinal injury.	^112^
Renal diseases	Acute kidney injury	Renal pedicles were clamped for 30 min	RIPK1^KD/KD^, RIPK3^−/−^, MLKL^−/−^	RIPK1 KD mutants, RIPK3, and MLKL deficiency could alleviate acute kidney injury by decreasing necroptosis.	^55^, ^113^, ^114^
		Kidney allograft rejection	RIPK3^−/−^	RIPK3 deficiency could preserve the function of kidney allografts.	^115^
	Chronic kidney disease	Subtotal nephrectomy surgery	RIPK3^−/−^, MLKL^−/−^, Nec‐1	Renal function and renal pathologic changes were significantly improved after RIPK3 or MLKL deficiency or administration of Nec‐1.	^53^, ^116^
		Diabetic nephropathy	None	Cassia auriculata ethanol leaf extract could improve the renal dysfunction and pathophysiology by inhibiting RIPK1/RIPK3.	^91^
Joint diseases	Rheumatoid arthritis	Collagen	Nec‐1	RIPK1, RIPK3, and pMLKL were significantly upregulated in vivo and vitro. Nec‐1 could protect articular cartilage injury.	^118^
	Osteoarthritis	ACLT	Nec‐1	The rats were protected from trauma‐induced cartilage degradation and limb pain by Nec‐1.	^121^
		DMM surgery	RIPK3^−/−^	RIPK3 depletion reduced OA pathogenesis.	^122^
Cancers	AML	Tumor‐bearing mice	RIPK3‐KD	The worse prognosis was associated with downregulated of RIPK3.	^141^
Ocular diseases	Glaucoma (Retinal I/R injury)	Infusion into the anterior chamber	Nec‐1	RIPK3 expression was rapidly increased in the mice retinas I/R. Nec‐1 could protect the histoarchitecture and thickness of the inner retina.	^147^, ^149^
	AMD	NaIO3, sodium iodate, idoacetic acid	Nec‐1	RIPK1 inhibitor could prevent RPE cells from necroptosis in retinal degeneration.	^150^, ^151^
	Retinitis pigmentosa	Rd10 mouse model	RIPK3^−/−^	RIPK1 and RIPK3 were increased in the degenerative retina. RIPK3 deficiency reduced cone cell death.	^152^
Infections	Viral infections	Vaccinia virus, Influenza A virus, West‐Nile virus, HSV‐1	RIPK3^−/−^	RIPK3‐/‐ mice were much more susceptible to viral infection. Necroptosis could result in the loss of CD4^+^ T cells.	^154^, ^157^, ^158^
		HIV	Nec‐1	Nec‐1 could protect CD4^+^ T from the damage of HIV in cell lines. RIPK3 silencing could restore the proliferation potential of CD8^+^ T cells from HIV‐infected patients. The necroptosis of CD4^+^ T cells was induced by Pmlkl	^159^, ^160^, ^161^
		COVID‐19	None	Inhibiting necroptosis protected mice from pathology and death induced by TNF‐α and IFN‐γ in COVID‐19 infected.	^162^
		CMV, HSV	None	CMV and HSV encoded caspase‐8 inhibitors to promote necroptosis.	^163^
	Bacterial infections	Salmonella	None	The suppression of necroptosis mediated by caspase‐8 reduced inflammatory damage in intestinal *Salmonella* infection.	^172^
		EPEC	None	EspL decreases the expressions of RIPK1, RIPK3, and TRIF leading to restricting necroptosis during infection	^173^
		Francisella	None	Francisella was proved to induce necroptosis	^174^
	Parasite infections	Oral T. gondii	RIPK3^−/−^	Deletion of RIPK3 could significantly improve survival after infection	^176^
		*Angiostrongylus cantonensis*	None	RIPK3, pRIPK3, pMLKL, and TNF‐α were increased by activated microglia caused the necroptosis of neurons.	^49^
Skin diseases	Vitiligo	Melanocyte	None	Oxidative stress induced melanocyte death through the RIPK1 signaling pathway.	^177^
	Psoriasis	Imiquimod	Nec‐1, NSA	Inhibition of RIPK1 or MLKL could reduce inflammation of psoriasiform dermatitis in mice.	^178^

ACLT, anterior cruciate ligament transection; AD, Alzheimer's disease; AMD, age‐related macular degeneration; AML, acute myeloid leukemia; ARDS, Acute respiratory distress syndrome; ARHL, Age‐related hearing loss; ALS, Amyotrophic lateral sclerosis; CMV, Murine cytomegalovirus; COPD, Chronic obstructive pulmonary disease; DMM, destabilisation of the medial meniscus; EPEC, enteropathogenic *Escherichia coli*; HIV, Human immunodeficiency virus; HSV, herpes simplex virus; IOP, intraocular pressure; IPF, Idiopathic pulmonary fibrosis; I/R, ischemia‐reperfusion; LADCA, left anterior descending coronary artery; MS, multiple sclerosis; PD, Parkinson's disease; RSV, respiratory syncytial virus.

Necroptosis is also involved in ischemia‐reperfusion injury (IRI)‐induced AKI has necroptosis. Compared with wild‐type mice, the level of RIPK1/3 in the heart of ischemic mice is significantly higher. In the acute IRI mouse model, RIPK3 deletion protects the heart from necroptosis induced by IRI and reduces the area of myocardial infarction (MI). Nec‐1 could protect heart from both the short‐ and long‐term effects of myocardial ischemia by reducing cell necroptosis and the area of MI and maintaining long‐term heart function.^33^ Meanwhile, inhibition of RIPK1 expression can block ischemia‐induced astrocyte necrosis^49^ (Tables 1, 2).

In liver, lung, kidney, and intestinal diseases, blocking necroptosis can reduce inflammation and contribute to reducing damage and protecting organs. In alcoholic hepatitis, RIPK3 increases after drinking, whereas RIPK3 deficiency can protect the liver from alcohol‐mediated damage by reducing the steatosis and inflammatory effects of ethanol on liver cells.^50^ In chronic obstructive pulmonary disease (COPD), Nec‐1 can reduce inflammation. In airway epithelial cells (AECs), endoplasmic reticulum chaperone protein GRP78 promotes smoking‐induced inflammation, which may be related to the upregulation of necroptosis and the NF‐κB pathway. Necroptosis mediates inflammatory response of macrophages induced by cigarette smoke (CS).^51^ In acute lung injury (ALI), necroptosis in hypoxia‐induced neonatal lung injury mice can be weakened by the deletion of the RIPK3 gene. RIPK3 deficiency mice survive form ventilator‐induced lung injury by significantly reducing systemic and pulmonary inflammation.^52^ From AKI to chronic kidney disease (CKD), under the regulation of IRI, the expression and interaction of RIPK3 and MLKL can induce necrosis of proximal renal tubular cells and promote the activity of inflammasome.^53^ Knocking out RIPK3 or MLKL protects renal tubular cells from necroptosis and inflammatory activation and prevents renal interstitial fibrosis after IRI. Necroptosis is activated in a TLR4‐dependent manner in the intestine of mice with necrotizing enterocolitis (NEC) and specifically upregulated in differentiated epithelial cells of immature ileal. Genes and drugs inhibiting necroptosis reduce mucosal inflammation and intestinal epithelial cell death in experimental NEC^54^ (Tables 1, 2).

**FIGURE 3:**
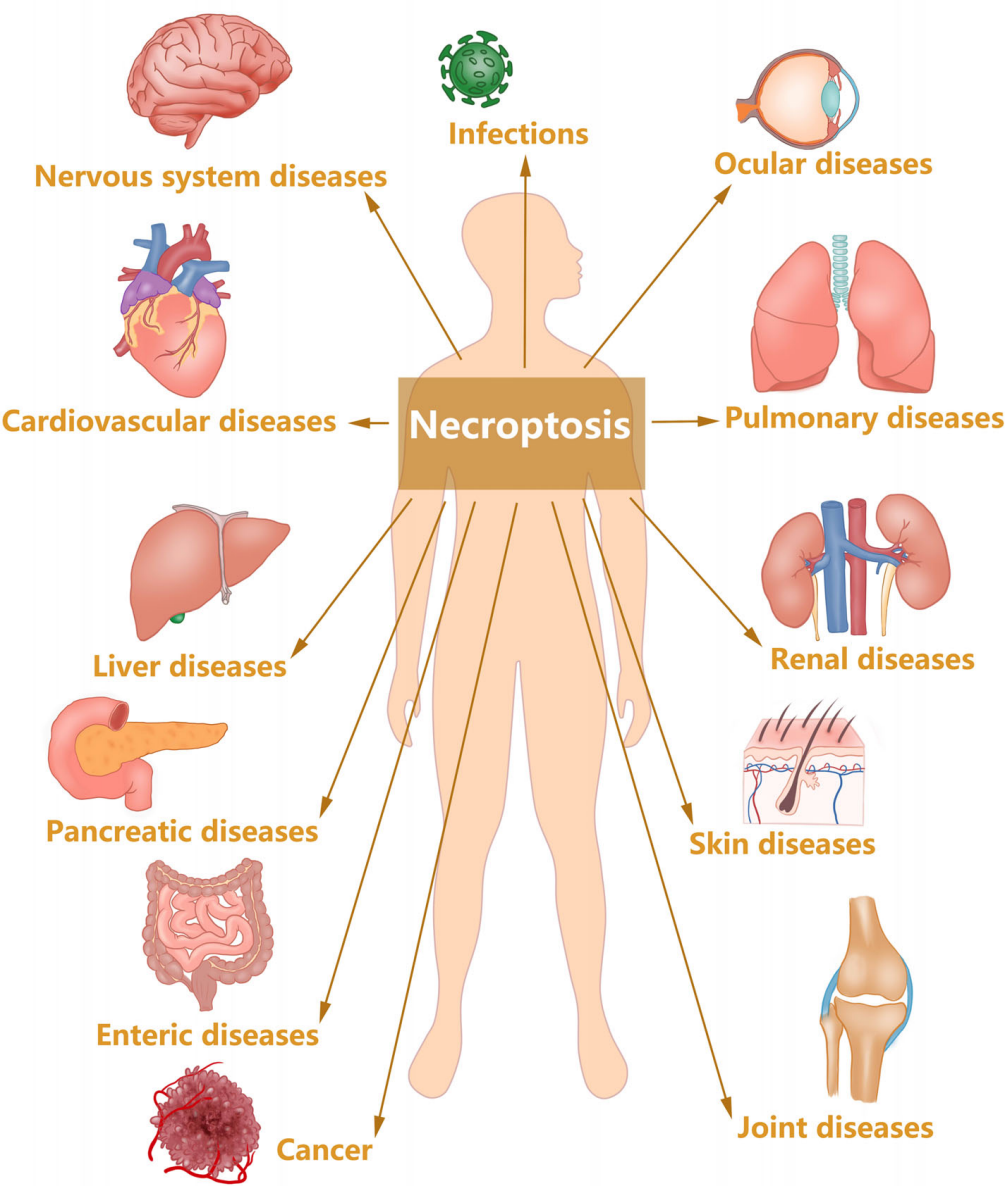
Necroptosis is involved in a verity of diseases, including infections, neurodegenerative diseases, cardiovascular diseases, cancers, pulmonary diseases, digestive system disease, renal diseases, joint diseases, ocular diseases, and skin diseases

The above is a brief description of necroptosis' role in tissue homeostasis, cancer, IRI, and inflammation. Necroptosis is involved much more than these including infections, neurodegenerative diseases, cardiovascular diseases, digestive system disease, pulmonary diseases, renal diseases, joint diseases, ocular diseases, skin diseases, and cancers (Figure 3). Details will be discussed as below.

## 2 NECROPTOSIS IN HUMAN DISEASES AND ANIMAL MODELS

### 2.1 Nerve system diseases

#### 2.1.1 Necroptosis in IRI

Ischemic stroke is a devastating brain injury usually resulting from thrombus/emboli within the cerebrovascular system. Degterev et al. provided the first example of active necrotic cell death in vivo and demonstrated that necroptosis plays a key role in the delayed ischemic brain injury following middle cerebral artery occlusion. Furthermore, the infarct volume was significantly reduced after the administration of Nec‐1.^4^ Interestingly, Newton and his colleagues confirmed that loss of RIPK3 had no effect on hypoxia‐induced cerebral edema or infarct volume in the major cerebral artery occlusion stroke model.^55^ But, Zhang et al. indicated that necroptosis played a crucial role in acute ischemic stroke. RIPK1 kinase‐dead mutants, deficiency of RIPK3 or MLKL could prevent acute ischemic stroke through blocking necroptosis. Together these data emphasize that RIPK1 inactivation (Nec‐1 inhibitor), RIPK3 deficiency, or MLKL deficiency should be examined to support the role of necroptosis in disease. Furthermore, inflammation is also a pathologic change that occurs after a stroke, the activity of RIPK1 and RIPK3 is associated with the activation of inflammatory‐related signal pathways in acute ischemic stroke.^56^ Inhibition of necroptosis could reduce cell death, meanwhile making the inflammatory response to be a protective reaction. Yang et al. showed that ischemia induced a neuron‐dominated necroptosis through the RIPK3/MLKL pathway. Ablating RIPK3 or MLKL could activate the microglia/macrophages from M1type, which expresses inflammatory factors and helps to remove dead cells and clear tissue debris, to the M2 type, which expresses anti‐inflammatory and growth factors to repair the damage of brain tissue in the ischemic cortex.^57^

#### 2.1.2 Parkinson's disease

Parkinson's disease (PD) is one of the most important neurodegenerative disorders associated with the progressive degeneration of dopaminergic neurons in the substantia nigra and depletion of dopamine release in the striatum. Previous studies indicated that necroptosis was activated in postmortem brain tissue of PD patients and a toxin‐based mouse model of PD. Expressions of RIPK1, RIPK3, and MLKL were elevated in the substantia nigra of PD‐derived postmortem tissue.^58^ Wu et al. found that Nec‐1 could provide protection and increase PC12 cell viability in PD models.^59^ Inhibition of RIPK1 and genetic ablation of MLKL and RIPK3 could decrease dopaminergic neuron degeneration in preclinical models of PD.^60^ Altogether, these studies support the idea that necroptosis may be a new target to prevent axonal degeneration in PD.

#### 2.1.3 Alzheimer's disease

Alzheimer's disease (AD) is a devastating neurodegenerative disease with no effective therapies so far. A previous study has found that the expressions of RIPK1, MLKL, and pMLKL were significantly increased in human AD brains compared to their age‐matched control counterparts.^61^ Accordingly, the levels of phosphorylated RIPK1, RIPK3, and MLKL were markedly higher in the AD rats compared to the control littermates. Treatment with RIPK1 inhibitor (Nec‐1)^62^ and MLKL inhibitor (NSA)^63^ could suppress necroptosis in AD brain. It further confirmed that necroptosis drives neuron death in AD.

#### 2.1.4 Amyotrophic lateral sclerosis

Amyotrophic lateral sclerosis (ALS) is a progressive neurodegenerative disease of the motor system characterized by the progressive loss of motor neurons in the brain and spinal cord, which leads to muscle weakness and eventual paralysis. Researchers have confirmed that the mechanism of neurodegeneration involves necroptosis in both mouse mutant SOD1 and the human ALS in vitro disease models.^64^ By pharmacological and genetic interventions targeting RIPK1, it confirmed that manipulating the necroptosis pathway could prevent motor neurons demise. Moreover, Ito et al. showed that the expressions of RIPK1, RIPK3, and pMLKL were elevated in the optineurin (a gene implicated in both familial and sporadic ALS) deficient mice. Therefore, treatment targeting these proteins normalized motor functions and improved axonal degeneration.^65^

#### 2.1.5 Multiple sclerosis

Multiple sclerosis (MS), is an autoimmune demyelinating and neurodegenerative disorder of the central nervous system (CNS). It is the leading cause of neurologic disability in young adults with no known cure. Ofengeim et al. found that the necroptosis markers, RIPK1, RIPK3, and MLKL, were activated in the cortical lesions of human MS pathological samples.^66^ Activation of necroptotic signaling often occurred in macroneurons in cortical layers II–III, but little expression in other cell types.^67^ All these findings show that necroptosis represents a new therapeutic target in MS treatment. Inhibition of RIPK1 could protect against oligodendrocyte cell death^66^ and attenuate demyelination^68^ in the animal models of MS.

#### 2.1.6 Age‐related hearing loss

Age‐related hearing loss (ARHL) is the most prevalent progressive neurodegenerative disorder in the elderly and is presently untreatable. Previous studies have shown that the levels of necroptosis markers, including RIPK1, RIPK3, and MLKL, were significantly elevated in aged mice compared to young mice. Considering all of this evidence, it seems that necroptosis plays a crucial role in cochlear aging in vivo.^69^

### 2.2 Cardiovascular diseases

#### 2.2.1 Myocardial infarction

MI is a life‐threatening emergency, caused by the blockage of coronary artery. Luedde et al. first proved that the RIPK3 expression was elevated in ischemic portions of mouse hearts undergoing left anterior descending coronary artery ligation.^70^ Deficiency of RIPK3 could diminish inflammatory response and reduce the infarct size in an acute I/R model, even have a long‐term cardioprotective effect.^33^, ^70^ Along a similar line, administration of Nec‐1 also has a cardioprotective by lessening the death of myocardial cell and infarct size in I/R injury.^71^, ^72^, ^73^ Therefore, inhibition of necroptosis may be amenable as a potential MI therapeutic modality.

#### 2.2.2 Aortic aneurysms

Aortic aneurysms are a common vascular disease in Western populations virtually involving any portion of the aorta. The most common locations are the infrarenal abdominal and ascending aortic regions in human. Aortic aneurysms are manifested as the progressive dilation with a high risk for death due to rupture. Wang and his colleagues observed that the expressions of RIPK1 and RIPK3 were elevated in human abdominal aortic aneurysm, especially medial smooth muscle cells, and the level of RIPK3 was also elevated in the mouse model of abdominal aortic aneurysm. Overexpression of RIPK3 could trigger SMC necroptosis while deletion of RIPK3 could impair inflammatory gene expression in aortic SMCs.^74^ Several studies have found that both Nec‐1 and GSK074, the necroptosis inhibitors, attenuate the aortic expansion in mouse models of abdominal aortic aneurysm.^75^, ^76^ Therefore, these results indicate that inhibiting necroptosis pathway represents a potential strategy for treating aortic aneurysms.

#### 2.2.3 Atherosclerosis

Atherosclerosis is a chronic inflammatory disease of the arterial wall, leading to acute thrombus and subsequent MI or stroke, and is a leading cause of cardiovascular mortality. Karunakaran et al. confirmed that the levels of RIPK3 and MLKL were higher in patients with unstable carotid atherosclerosis. Low‐density lipoprotein promotes RIPK3 and MLKL transcription and phosphorylation. Nec‐1 could reduce the lesion size and markers of plaque instability in mice model. Collectively, these findings suggest that necroptosis can be targeted as a therapeutic tool for treating unstable atherosclerosis.^77^

#### 2.2.4 Diabetic cardiomyopathy

Chen et al. demonstrated that necroptosis was involved in diabetic cardiomyopathy. Deficiency of RIPK3 mitigated myocardial injury, improved cardiac function, suppressed CaMK II activation, and reduced necroptosis in mice with diabetic cardiomyopathy mice. Moreover, CaMK II activation and necroptosis augment in diabetic cardiomyopathy were achieved via a RIPK3‐dependent manner. These findings may open novel avenues for therapeutic strategies against diabetic cardiomyopathy.^78^

### 2.3 Pulmonary diseases

#### 2.3.1 Chronic obstructive pulmonary disease

COPD is a chronic smoking‐related lung disease, characterized by persistent respiratory symptoms and progressive airflow obstruction, associated with significant morbidity and mortality, killing more than 3 million people worldwide every year.^79^ In recent years, there has been an increasing amount of literature that has shown that necroptosis plays a vital role in this fatal disease. Mizumura et al. demonstrated that necroptosis could potentially contribute to COPD pathogenesis through activation of RIPK3 and mitophagy induced by CS exposure. Furthermore, they also discovered the elevated expression of RIPK3 was in human COPD lung.^80^ Similarly, Lu et al. found that the total MLKL protein in the epithelium and macrophages along with the pRIPK3 and pMLKL in lung tissue were elevated in patients with severe COPD. Deficiency of RIPK3 or MLKL could attenuate CS‐induced airway inflammation, airway remodeling, and emphysema.^51^ In mice model, CS‐induced necroptosis and DAMP release could initiate neutrophilic airway inflammation in mice. Treatment with the necroptosis inhibitor Nec‐1 could attenuate neutrophilic inflammation induced by CS accordingly.^81^ In general, these findings indicate that necroptosis plays a pathogenic role in COPD and indicate that it could be a potential therapeutic target in COPD treatment.

#### 2.3.2 Acute respiratory distress syndrome

Acute respiratory distress syndrome (ARDS) is a syndrome of acute respiratory failure caused by noncardiogenic pulmonary edema, characterized by rapidly progressive dyspnea, tachypnea, and hypoxemia. ARDS is thought to occur when a pulmonary or extrapulmonary insult causes the release of inflammatory mediators, promoting inflammatory cell accumulation in the alveoli, and microcirculation of the lung. Previous studies have shown that the expressions of RIPK1, RIPK3, and MLKL were remarkably elevated in lung tissue from the rat model of ARDS.^82^, ^83^ Treatment with Nec‐1 could ameliorate pulmonary function dramatically in osteoarthritis (OA)‐induced ARDS rats. Furthermore, Nec‐1 could downregulate RIPK1‐RIPK3‐MLKL signal pathway and inhibit inflammatory response by reducing neutrophil infiltration and protein leakage in a rat model of ARDS.^83^ Interestingly, Wang et al. had shown that RIPK3 was significantly increased in the severe ARDS model, while accompanied pRIPK3 and MLKL were increased in the mild ARDS group. Knocking out RIPK3 attenuated the hypothermia symptom, ameliorated the lung tissue injury, and increased the survival rate.^84^

#### 2.3.3 Asthma

Asthma is a chronic heterogeneous disease of the lower airways characterized by chronic inflammation, airway remodeling, and airway hyper‐reactivity leading to cough, wheeze, breathing difficulties, and chest tightness.^85^ Previous studies have shown that eosinophil cytolysis takes place through RIPK3‐MLKL‐dependent necroptosis, which can be negatively regulated by autophagy.^86^ Treatment with GW806742X, a human and murine necroptosis inhibitor, blocked necroptosis and IL‐33 release in vitro and reduced eosinophilia in *Aspergillus fumigatus* extract‐induced asthma in vivo.^87^ In a mouse model of asthma exacerbations induced by in house dust mite for inflammation and double‐stranded RNA for exacerbation, RIPK3 and phosphorylation of MLKL were increased in IFN‐β‐/‐ mice, so the absence of IFN‐β may augment markers of necroptotic cell death at exacerbation.^88^ Particulate matter 2.5 μm is a well‐recognized risk factor for asthma. Zhao et al. have shown that PM2.5 could enhance airway hyperresponsiveness, which was induced by necroptosis‐related inflammation.^89^ Respiratory syncytial virus (RSV)‐associated bronchiolitis is the major cause of morbidity and mortality in infancy worldwide. And severe RSV‐bronchiolitis during infancy is associated with increased asthma risk in childhood. RSV infection induces the necroptosis of AEC. Inhibition of the RIPK1 and MLKL can ameliorate the severity of viral bronchiolitis in mice and prevent the later progression to asthma. The abovementioned results suggested that inhibition of necroptosis may be a new approach to limit the severity of bronchiolitis and break its nexus with asthma.^90^

#### 2.3.4 Idiopathic pulmonary fibrosis

Idiopathic pulmonary fibrosis (IPF) is a chronic and progressive idiopathic interstitial pneumonia, characterized by an abnormal fibrotic response involving vast areas of the lungs and ultimately respiratory failure and death. Lee et al. showed that the levels of RIPK3 and pMLKL were markedly raised in human IPF lungs, which indicated necroptosis might contribute to IPF development. RIPK3, HMGB1, and IL‐1β were also increased in the lung fibrosis models induced by bleomycin (BLM). Decreased BLM‐induced DAMPs secretion and attenuated lung inflammation and fibrosis were observed in RIPK3‐deficient mice model.^91^ In general, blockage of necroptosis may be a promising therapeutic approach for IPF.

#### 2.3.5 Acute lung injury

RIPK3‐mediated necroptosis was detected in the hypoxia‐induced lung injury of neonatal mice, which was attenuated by the deletion of RIPK3.^92^ Similarly, RIPK3‐mediated necroptosis also involved in the development of an LPS‐induced ALI mouse model. Inhibition of RIPK3 could significantly reduce LPS‐induced necroptosis and NLRP inflammasome activation accompanied by reduced production of IL‐1β and IL‐18 production and inflammatory cell infiltration.^52^ RIPK3‐deficient mice, but not MLKL deficiency, could protect the mice against ventilator‐induced lung injury in a fatty acid β‐oxidation‐dependent manner.^93^ Treatment with Nec‐1 could attenuate lung injury, systemic and lung inflammation, and improve survival rate in neonatal mice with sepsis.^94^

### 2.4 Liver disease

Liver diseases are the major causes of illness and death worldwide ranging from hepatitis B, hepatitis C, alcoholic liver disease (ALD), nonalcoholic fatty liver disease, hepatic fibrosis, cirrhosis, liver failure, and HCC.^95^

Liver injury is generally classified into two clinical subtypes by duration: acute and chronic. Acute liver failure, the most extreme form of acute liver injury, is a clinical syndrome occurred in patients without pre‐existing liver disease and characterized by coagulopathy and encephalopathy. Chronic liver injury is characterized by the presence of persistent liver damage and hepatitis from inflammation or intracellular stress responses.

Previous research has shown that phosphorylated MLKL translocated to the plasma membrane and then executed cell death.^8^, ^96^ This process is driven by RIPK3‐dependent phosphorylation and is the necessary step of necroptosis. Hence, RIPK3 and MLKL are considered to be the important components of activated necroptosis.^97^ It is worth noting that the expression of RIPK3 is absent under basal conditions, which is contrary to MLKL.^98^ Based on our knowledge, hepatocytes should be not susceptible to necroptosis due to the absence of RIPK3 in hepatocytes.^99^ However, under a certain condition, MLKL could induce liver cell death in a RIPK3‐independent fashion in immune‐mediated liver disease.^100^ Roychowdhury and his colleagues explored the effect of necroptosis in the different liver models. They found that RIPK3 expression was increased while RIPK1 expression had no change in liver tissues of both humans with ALD and mice after chronic feeding. RIPK3^–/–^ mice had less ethanol‐induced steatosis and hepatocyte injury compared to the controls group, but treatment with Nec‐1 did not protect against ethanol‐induced injury.^50^ They also observed that the levels of RIPK3 and MLKL were higher in liver injury in response to high‐fat diets. Interestingly, on contrary to the alcohol model, deficiency of RIPK3 could worsen hepatic steatosis, inflammation, and hepatocyte injury.^101^ Different from chronic liver injury, studies have found that RIPK1 phosphorylation was induced by acetaminophen in the mouse model of acute liver injury. Treatment with Nec‐1 could inhibit reactive oxygen species production in acetaminophen‐damaged hepatocytes.^102^ Ramachandran and his colleagues found that RIPK3 functioned as an early mediator of acetaminophen‐induced hepatocyte necrosis in mice. Inhibiting RIPK3 or using RIPK3‐deficient mice could attenuate necrotic cell death.^103^ Qian and his colleagues confirmed that mice undergo necroptosis by RIPK1‐RIPK3‐MLKL pathway upon *Listeria monocytogenes* infection, subsequently resulting in acute hepatic damage. RIPK1 deletion was found to significantly alleviate mitochondrial dysfunction and necroptosis in *Listeria monocytogenes*‐infected acute liver injury.^104^ In previous studies, treating with Nec‐1 or MLKL deficiency could protect the liver from Concanavalin A.^105^ Taken together, how necroptosis contributes to liver injury pathogenesis remains to be further explored.

### 2.5 Acute pancreatitis

Acute pancreatitis (AP) is a local inflammatory disease of the pancreas with multiple causes. The role of necroptosis in the pathophysiology of pancreatitis appears to be controversial because differences in the contribution of necroptosis to the AP have been reported. Inhibition of Nec‐1 or knockout of either RIPK3 or MLKL exhibited a reduction in cell death in the pancreas on induction of pancreatitis.^106^, ^107^, ^108^, ^109^, ^110^ In contrast, Boonchan et al. found that RIPK3‐ or MLKL‐deficient mice experienced more severe pancreatic edema and inflammation than control mice.^111^ It suggests that necroptosis mediated by RIPK3 and MLKL have a protective effect on AP, whether to use inhibitors of necroptosis requires careful consideration for AP treatment.

### 2.6 Enteric diseases

#### 2.6.1 Necrotizing enterocolitis

NEC is the leading cause of death to gastrointestinal disease in premature infants, which can have devastating effects. Researchers have shown that necroptosis pathway genes, including RIPK1, RIPK3, and MLKL, were significantly upregulated in both NEC patients tissue and established mouse model of NEC. Upregulation of necroptosis is positively correlated with the degree of intestinal damage. Necroptosis inhibition by either genetic or chemical intervention could relieve the intestinal damage, indicating that necroptosis plays an important role in the NEC‐induced intestinal damage. Strikingly, the addition of human breast milk oligosaccharide 2 fucosyllactose could reduce necroptosis and inflammation.^54^

#### 2.6.2 Intestinal injury

Previous studies have shown that damage to the small intestine can be a major contributor to the morbidity and mortality associated with heat stroke. Heat Stress elevated the levels of RIPK1, RIPK3, and phosphorylated MLKL, leading to form the necrosome both in vivo and in vitro. Pretreated with the Nec‐1 or GSK872 could significantly reverse these phenomena. These findings provide the basis for developing new therapeutic strategies for patients diagnosed with heat stroke.^112^

### 2.7 Renal diseases

#### 2.7.1 Acute kidney injury

AKI is defined by a rise in serum creatinine, decreased urine output and accumulation of metabolic toxins, resulting in an abrupt reduction in kidney function. Several studies have suggested that inhibition of Nec‐1 or deficiency in RIPK3 exerts protective effects in AKI caused by IR injury.^113^, ^114^ Lau A found that RIPK3 deficiency preserved the function of kidney allografts.^115^ Similarly, MLKL deficiency was protective against intrinsic renal injury, but not in intrinsic prerenal injury.^55^

#### 2.7.2 Chronic kidney disease

Necroptosis was also found in chronic kidney injury of subtotal nephrectomized rats. Zhu et al. found that necroptotic cell death and the highest level of RIPK1 and RIPK3 appeared 8 weeks after subtotal nephrectomy surgery. The renal function and renal pathologic changes improved after administration of Nec‐1 and the overexpression of RIPK1, RIPK3, MLKL was reduced.^116^ Chen et al. showed that necroptosis of renal proximal tubular cells was activated by RIPK3 and MLKL under the conditions of IRI during AKI to CKD process. Knockdown of RIPK3 or MLKL could attenuate renal tubular cell necroptosis, macrophage infiltration, and then ultimately prevent the progression of interstitial fibrogenesis after IRI.^53^

### 2.8 Joint diseases

#### 2.8.1 Rheumatoid arthritis

Rheumatoid arthritis (RA) is an autoimmune disease characterized by chronic and systemic inflammation. RA is associated with articular cartilage and bone erosion, synovitis, joint deformity, reduced quality of life, and increased mortality. Necroptosis plays a crucial role in systemic inflammatory response syndrome including RA.^117^ Chen et al. further confirmed the involvement of necroptosis in articular chondrocytes of the RA model. The expressions of RIPK1, RIPK3, and pMLKL were markedly elevated both in the adjuvant arthritis rat articular cartilage in vivo and in acid‐induced chondrocytes in vitro. Treatment with Nec‐1 could inhibit chondrocyte necroptosis and prevent articular cartilage damage and reduce the amount of proinflammation cytokine.^118^ Overall, these results indicate that pharmacological blockade of necroptosis could be a potential therapeutic approach for RA treatment.

#### 2.8.2 Osteoarthritis

OA, the most common degenerative disease characterized by articular cartilage destruction, is caused by mechanical stress, abnormal joint mechanics, and multiple risk factors. According to recent reports, significant accumulation of oxidative stress and cytokine release in OA cartilage, implies a complex relationship between cartilage injury and necroptotic processes.^119^, ^120^ Cheng et al. found that RIPK1 expression was markedly increased in OA cartilage, both in OA patients and experimental rat models. Upregulation of RIPK1 in articular cartilage could induce structural and functional defects of cartilage in rats, indicating the vital role of RIPK1 in disrupting extracellular matrix metabolism homeostasis and regulating chondrocyte necroptosis during OA progression. The RIPK1 kinase inhibitor (Nec‐1) could potently prevent the trauma‐induced cartilage degradation and limb pain in rats.^121^ Consistent with RIPK1, RIPK3 also plays a crucial role in OA pathogenesis. Jeon et al. found that RIPK3 expression was markedly higher in OA‐damaged regions of human cartilage than in samples of undamaged cartilage. RIPK3 overexpression could accelerate cartilage disruption in the mouse model, whereas RIPK3 deficiency or inhibition of RIPK3 attenuates OA.^122^ Altogether, these results provide new insights into the pathogenesis of OA and open new therapeutic avenues for treating OA.

### 2.9 Cancers

The role of necroptosis in cancer remains controversial because it can be both a tumor suppressor and a tumor promoter.^123^, ^124^ Necroptosis could induce abnormal cells death leading to a good prognosis, while it could also lead to inflammation and cancer metastasis.^125^, ^126^ Upregulation and downregulation of major necroptotic members have been observed in many types of cancers. The expression of RIPK1 is generally maintained at a normal level in a variety of cancers, but its expression is upregulated in glioblastoma, lung cancer, and MDS, which has a negative impact on cancer prognosis.^125^, ^127^, ^128^, ^129^ In addition to RIPK1, RIPK3 and MLKL are also involved in oncogenesis and related to prognosis. RIPK1/3 or MLKL is downregulated in breast cancer,^130^, ^131^ colorectal cancer,^132^, ^133^, ^134^ melanoma,^135^ gastric cancer,^136^ ovarian cancer,^137^ head and neck squamous cell carcinoma,^138^ cervical squamous cell carcimoma,^139^ and AML.^140^, ^141^ Since necroptosis can function as a tumor suppressor or a tumor promoter, the role of necroptosis in different types of malignant tumors remains to be further studied.

Similarly, necroptosis may be a double‐edged sword at different stages of cancer metastasis. The tumor cells spread mostly through the circulatory system to colonize distant organs in the early stage of metastasis. In this process, endothelial cell necroptosis induced by tumor cells could promote extravasation and metastasis.^47^ Dong et al. has found that depletion of MLKL effectively inhibits invasion of radio‐resistant NPC by suppressing epithelial‐mesenchymal transition. MLKL may be a promising target to suppress distant metastasis of NPC patients who relapsed after radiotherapy.^142^ Inhibitors targeting RIPK1 activity could significantly repress metastasis of both lung carcinoma cells and melanoma cells in mice.^143^ Administration of aurora kinase inhibitor could induce necroptosis and immunogenic cell death, inhibit tumor growth, and reduce metastasis in mice PANC1 subcutaneous tumor models.^144^ Han et al. found that Resibufogenin could suppress the growth and metastasis of colorectal cancer through induction of RIPK3‐dependent necroptosis in vivo.^145^ Thus, further research is urgently required to explore the complex relationship between necroptosis and cancer progression and metastasis.

### 2.10 Ocular diseases

#### 2.10.1 Glaucoma

Glaucoma is a group of chronically progressive diseases of optic nerve, which causes degeneration of retinal ganglion cells (RGC) and peripheral visual field loss. Previous findings have shown that both ischemia and hypoxia could induce necroptosis of RGCs. Nec‐1 has an apparent neuroprotective effect on RGC in culture.^146^ In the mouse model of retinal IRI for in vivo and the RGC cell model of oxygen and glucose deprivation for in vitro, Nec‐1 could significantly reduce the retinal damage caused by ischemia‐reperfusion, increase RGC survival, and attenuate proinflammatory response in the ischemic retina.^147^ In addition to RIPK3, it has been shown that the expression of RIPK3 is rapidly increased in the ganglion cell layer and the inner nuclear layer of the mice retinas following acute increases in intraocular pressure^148^ and ischemia‐reperfusion.^149^ These findings indicate that necroptosis appears to be responsible for the development of glaucoma and the inhibition of necroptosis components may be a novel disease therapeutic approach for treating glaucoma.

#### 2.10.2 Age‐related macular degeneration

Age‐related macular degeneration (AMD) is a degenerative disease of the macula with visual impairment, which is the leading cause of blindness in the elderly. The etiology of AMD is unclear due to the involvement of numerous factors, among which oxidative stress plays a key role. Researchers have found that retina pigment epithelial (RPE) cells necroptosis is related to RPE cells death under oxidative stress in vitro. Treatment with Nec‐1 or downregulation of RIPK3 by siRNAs could rescue necroptosis induced by oxidative stress. Meanwhile, RPE necroptosis in a retina degeneration mouse model induced by NaIO_3_ is also supported by the use of Nec‐1.^150^ Jang et al. revealed that RIPK1 inhibitor prevented RPE cells from necroptosis in retinal degeneration in sodium iodate‐injected rabbits and iodoacetic acid‐treated mini pigs.^151^ Murakami et al. found that RIPK1 and RIPK3 were accumulated in the degenerative retina and RIPK3 deficiency reduced cone cell death.^152^ Collectively, necroptosis is a major form of RPE cell death in response to oxidative stress and it has the potential to provide a novel therapeutic rationale for AMD.

### 2.11 Pathogen invasion

#### 2.11.1 Viral infections

Necroptosis is a host defense mechanism against viral infection.^153^ For example, RIPK3^–/–^ mice infected with vaccinia virus could exhibit severely impaired virus‐induced tissue necrosis, inflammation, and control of viral replication.^5^ RIPK3^–/–^ mice were highly susceptible to influenza A virus infection, precisely because RIPK3‐deficient macrophages could not produce type I IFN. As a result, the infected mice exhibited elevated pulmonary viral load and heightened morbidity and mortality.^154^ Studies have observed that mice with deficiency of ZBP1 or RIPK3 had high lung virus titers and hypersusceptible to lethal IAV infection.^154^, ^155^ Zhang et al. found that Z‐RNAs produced by replicating IAV‐activated ZBP1, which activated MLKL to further lead to necroptosis and nuclear envelope rupture in an “inside‐out” pathway. MLKL‐activated nuclear envelope rupture during IAV infection releases nuclear DAMPs, served as a potent stimulator of neutrophils in cell culture. Consequently, the recruitment and activation of neutrophils is promoted in the infected lung contributing to the severity of IAV disease in vivo.^156^ RIPK3 and its interaction with the herpes simplex virus (HSV)‐1protein ICP6 could trigger the cells necroptosis in infected mouse cells and limit viral propagation in mice.^157^ RIPK3^–/–^ mice infected with West Nile virus exhibited enhanced mortality by suppressing neuronal chemokine expression and decreasing CNS recruitment of T lymphocytes and inflammatory myeloid cells.^158^

However, it is noteworthy that HIV‐1 could induce necroptosis in the infected primary CD4^+^ T lymphocytes and CD4^+^ T‐cell lines, leading to the progressive loss of CD4^+^ T cell. Treatment with Nec‐1 potently restrained HIV‐1‐induced cytopathic effect and inhibited the formation of HIV‐induced syncytia in CD4^+^ T‐cell lines.^159^ Other studies have also confirmed this conclusion through in vivo experiments. Gaiha et al. shows that blockade of necroptosis and silencing of RIPK3 expression could restore the proliferation potential of CD8^+^ T cells from HIV infected patients.^160^ Terahara et al. identified that X4 HIV‐1 induced necroptosis characterized by pMLKL expression in CD4^+^ T cells.^161^ Karki et al. found that the combination of TNF‐α and IFN‐γ induced inflammatory cell death characterized by pyroptosis, apoptosis, and necroptosis during SARS‐CoV‐2 infection. TNF‐α and IFN‐γ caused a lethal cytokine shock in mice reflecting the tissue damage and inflammation of COVID‐19, and inhibiting necroptosis protected mice from this pathological condition and death.^162^ Murine cytomegalovirus (CMV) and HSV encode caspase‐8 inhibitors, which could prevent apoptosis together with competitors of RHIM‐ dependent signal transduction to interrupt the necroptosis. Human CMV blocks TNF‐α‐induced necroptosis after RIPK3 activation and phosphorylation of the pseudokinase MLKL, which is different from RHIM signaling competition by murine CMV or HSV.^163^

#### 2.11.2 Bacterial infections

Most studies have shown that necroptosis has a detrimental effect on bacterial infections. Necroptosis of immune cells, such as neutrophils and macrophages, induced by *Staphylococcus aureus* often impeded host defense, leading to tissue damage or even death. Blockage of necroptosis could enhance the clearance of *S. aureus*, decrease lung tissue injury, and forestall the progression of pneumonia.^164^, ^165^ González‐Juarbe et al. proved that various bacterial pathogens, inclusive of *Streptococcus marcescens*, *Listeria monocytogenes*, *S. aureus*, uropathogenic *Escherichia coli*, *Streptococcus pneumonia* (SPn), that produce a pore‐forming toxin (PFT) induce macrophages necroptosis, which could be blocked for defending toward death by inhibiting of RIPK1, RIPK3, and MLKL.^166^ Jondle et al. first reported that *Klebsiella pneumonia* (KPn) impair the neutrophil efferocytosis through activation of necroptosis pathway improving the disease outcome in‐vivo in a preclinical mouse model of KPn pneumonia through treatment of KPn‐infected mice with Nec‐1 or GSK’872 (RIPK3 inhibitor).^167^ Interestingly, using a mouse model of SPn asymptomatic colonization, Riegler et al. found that PFT‐induced necroptosis contributed to the natural development of protective immunity against opportunistic PFT‐producing bacterial pathogens.^168^ In human studies, RIPK3 protein concentrations were significantly elevated and were identified as a potential plasma marker of pneumococcal pneumonia. RIPK3‐initiated necroptosis is essential for host defense against *S. pneumonia*.^169^ Activation of TLR/TNF signaling during infection, combined with multiple expressions of apoptosis‐regulatory proteins, could also promote necroptosis indicating the phosphorylation of MLKL during BMDM infection.^170^, ^171^ It is noteworthy that the inhibition of caspase‐8‐mediated necroptosis seems to be important to limit excessive inflammatory damage to the host during intestinal *Salmonella* infection.^172^ Enteropathogenic *E. coli* (EPEC) uses the type III secretion system effector EspL to degrade the RHIM‐containing proteins RIPK1, RIPK3, TRIF, and ZBP1/DAI leading to restricting necroptosis and pyroptosis during infection.^173^ The highly pathogenic Francisella has been shown to cause necrosis and the highly virulent species of *Francisella* could induce necroptosis. However, the molecular mechanisms underlying remain to be clarified.^174^

#### 2.11.3 Parasite infections

Rosenberg and Sibley have shown that *Toxoplasma gondii* effector TgNSM, which targeted NCoR/SMRT complex, antagonizes the interferon to activate necroptosis. This process of *T. gondii* to prevent the necroptosis contributes to the survival of intracellular cycts and chronic infection.^175^ However, there is still uncertainty about the role of necroptosis in parasites killing or control in infected cells. Deletion of RIPK3 could significantly improve survival after oral *T. gondii* infection but do not increase the parasite burden.^176^ *Angiostrongylus cantonensis* infection might considerably elevate the levels of RIPK3, pRIPK3, and pMLKL, and in vitro assay discovered that TNF‐α secretion by activating microglia caused the necroptosis of neurons,^49^ providing a new therapeutic strategy for CNS injury.

### 2.12 Skin diseases

#### 2.12.1 Vitiligo

Vitiligo is a common autoimmune depigmenting skin disorder histologically characterized by the extensive destruction of epidermal melanocytes. Li et al. found that necroptosis was involved in the death of melanocytes through the RIPK1‐RIPK3‐MLKL pathway under oxidative stress in vitiligo. Accordingly, treatment with Nec‐1 or deficient MLKL could enhance the resistance of melanocytes to hydrogen peroxide‐induced necroptosis.^177^ Thus, targeting necroptosis may provide a new approach to the treatment of vitiligo.

#### 2.12.2 Psoriasis

Psoriasis is an autoimmune skin disorder characterized by hyperproliferation and maturation impairment of keratinocytes. Duan et al. found that MLKL and RIPK1 were markedly expressed in all layers of the epidermis in human psoriasis epidermis. Moreover, pMLKL and RIPK3 were primarily expressed in the upper layers. Inhibition of RIPK1 or MLKL could attenuate necroptosis both in vitro and in vivo, and powerfully reduce inflammation in psoriasiform dermatitis mice induced by IMQ. Therefore, inhibiting necroptosis can potentially provide a new target for the treatment of psoriasis.^178^

#### 2.12.3 Stevens–Johnson syndrome and toxic epidermal necrolysis

Stevens–Johnson syndrome (SJS) and toxic epidermal necrolysis (TEN) are life‐threatening diseases characterized by the death of keratinocytes. Both apoptosis and necroptosis contribute to keratinocyte death. Abundant RIPK3 and pMLKL expressing keratinocytes could be detected in the epidermis of SJS/TEN skin lesions. Furthermore, the levels of serum RIPK3 were elevated in the acute phase of patients with SJS/TEN. All these results suggested that serum RIPK3 levels could be used as a biomarker of the diagnostic and severity of SJS/TEN.^179^

## 3 DRUGS AND AGENTS THAT REGULATE NECROPTOSIS

Necroptosis is a very important pattern of cell death. It is not merely involved in the maintenance of organismal homeostasis, but also plays a vital role in the regulation of disease occurrence and development.^45^ Necroptosis is a double‐edged sword in diseases and its development. Researches have shown that inhibiting necroptosis is meaningful for maintaining internal homeostasis and restraining inflammation, ischemia, and toxic syndromes.^45^, ^180^ Meanwhile, the necroptosis induced by drugs and agents was used as a therapeutic paradigms in the treatment of many tumors. Hence, drugs that inhibit or promote necroptosis may have therapeutic potential for human disease (Table 3).

**TABLE 3:** The induce and inhibit drug or agentia

Drugs or agentia		Disease	Target	References
Induce	PFK15	Colorectal cancer cells	RIPK1, RIPK3, and MLKL	^181^
	ABT737	Bladder cancer	RIPK3 and MLKL	^184^
	Smac mimetic BV6	Burkitt's lymphoma, pancreatic carcinoma	Mlkl	^182^, ^183^
	Obatoclax	Different cancers	RIPK1, RIPK3, and MLKL	^185^
	Interferons	Different diseases	RIPK3 and MLKL	^186^
	Ethanol	Gastric epithelial cells	RIPK1	^187^
	Valproic acid	Epilepsy and mood disorders	RIPK1	^188^
	Carbon ion	Nasopharyngeal carcinoma	MLKL	^189^
	Decitabine	Breast cancer	RIPK3	^190^
	5‐azacytidine	Breast cancer	RIPK3	^190^
	5‐fluorouracil	Different cancers	RIPK1 and RIPK3	^191^, ^192^
	Cisplatin	Different cancers	RIPK1, RIPK3, and MLKL	^193^, ^194^
	Anthracyclines	Lung cancer	RIPK3 and MLKL	^195^
	Oxaliplatin	Lung cancer	RIPK3 and MLKL	^195^
	Tanshinone	Hepatocellular carcinoma	RIPK1 and RIPK3	^196^
	Shikonin	Pancreatic, osteosarcoma, prostate cancer, and non‐small cell lung cancers	RIPK1 and RIPK3	^197^, ^198^
	Emodin	Renal cancer	RIPK1, RIPK3, and MLKL	^199^, ^200^
	Bufalin	Pancreatic and breast cancers	RIPK1 and RIPK3	^201^
	Resibufogenin	Pancreatic and colorectal cancers	RIPK3 and MLKL	^145^
	Curcumin	prostate carcinoma	RIPK3 and MLKL	^202^
	Neoalbaconol	Nasopharyngeal carcinoma	RIPK1 and RIPK3	^203^
Inhibit	Dabrafenib	Melanoma	RIPK3	^204^
	Carfilzomib	Multiple myeloma	RIPK3 and MLKL	^205^
	Sorafenib	Hepatocellular, thyroid, and renal cell cancers	RIPK1 and RIPK3	^206^
	Pazopanib	Renal cell carcinoma and advanced soft tissue sarcoma	RIPK1	^207^
	Ponatinib	Leukemia, ischemic heart injury	RIPK1 and RIPK3	^208^
	ZYZ‐803	Acute myocardial ischemia	RIPK3	^209^
	Necrostatin‐1	hypertrophic scars	RIPK1	^210^
	GSK2982772	Rheumatoid arthritis, colitis, and psoriasis	RIPK1	^211^
	GSK3145095	Pancreatic cancer	RIPK1	^212^
	Necrosulfonamide(NSA)	Alzheimer's Disease, Amyotrophic lateral sclerosis	MLKL	^63^, ^64^
	GSK074	Aortic aneurysms	RIPK1 and RIPK3	^76^
	GW806742X	Asthma	MLKL	^87^
	SAR443820	Healthy Subjects	RIPK1	*
	SAR443122	Immune System in Severe COVID‐19, Cutaneous Lupus Erythematosus	RIPK1	*
	GFH312	Healthy Subjects	RIPK1	*
	Geldanamycin	Methamphetamine neurotoxicity, Cortical neurons	RIPK3 and MLKL	^216^, ^217^
	Geldanamycin	Pancreatic cancer	RIPK1	^215^
	Thio‐benzoxazepinones	Cells	RIPK1, RIPK3, and MLKL	^218^
	Bardoxolone derivatives 20	Cells	RIPK1 and RIPK3	^219^
	Cyclosporine A	Immunosuppressive drug	RIPK1 and RIPK3	^213^
	Rapamycin	Restenosis in coronary arteries, retinal detachment, and transplant rejection in lymphangioleiomyomatosis	RIPK1	^214^
	Phenytoin	Epilepsy and breast cancer	RIPK1	^184^
	Chiral benzothiazole	Cells	RIPK1, RIPK3, and MLKL	^220^
	Aucubin	Epilepsy	RIPK1 and MLKL	^221^
	Wogonin	Acute kidney injury	RIPK1	^222^
	Patchouli alcohol	Colitis	RIPK3 and MLKL	^223^
	Cassia auriculata leaf extract	Diabetic nephropathy	RIPK1 and RIPK3	^224^

* The website of clinical trials (https://clinicaltrials.gov).

Several small molecule inhibitors have been reported to induce expression of the key necroptosis molecules including selective 6‐phosphofructo‐2‐kinase (PFKFB3) inhibitor PFK15,^181^ Smac mimetic BV6,^182^, ^183^ Bcl‐2 inhibitor ABT737,^184^ and Obatoclax.^185^ Numerous drugs have also been proved to induced necroptosis, such as interferons,^186^ ethanol,^187^ and valproic acid.^188^ Carbon ion was found to induce the expression of MLKL in nasopharyngeal carcinoma.^189^ The chemotherapeutic drugs, including Decitabine, 5‐azacytidine, 5‐fluorouracil, cisplatin, anthracyclines, and oxaliplatin, are critical in cancer therapy by inducing necroptosis.^190^, ^191^, ^192^, ^193^, ^194^, ^195^ Moreover, traditional Chinese medicines, such as tanshinone,^196^ shikonin,^197^, ^198^ emodin,^199^, ^200^ bufalin,^201^ curcumin,^202^ and resibufogenin,^145^ also target necroptosis induction in the treatment of different tumors. Neoalbaconol, a naturally occurring small molecule compound, could inhibit the nasopharyngeal carcinoma by inducing necroptosis.^203^ To sum up, small molecule inhibitors, chemotherapeutic drugs, traditional Chinese medicines drugs, or other agents could be used to treat multiple diseases by inducing necroptosis.

On the contrary, it is also documented that a variety of drugs can downregulate necroptosis including small molecule inhibitors, immunosuppressive drugs, traditional Chinese medicines drugs, and other agents. There were plenty of small molecule inhibitors used in the treatment of multiple diseases or research in healthy subjects such as Dabrafenib,^204^ Carfilzomib,^205^ Sorafenib,^206^ Pazopanib,^207^ Ponatinib,^208^ ZYZ‐803,^209^ Nec‐1,^210^ GSK2982772,^211^ GSK3145095,^212^ NSA,^63^, ^64^ GSK074,^76^ and GW806742X.^87^ The immunosuppressive drug Cyclosporine A^213^ and Rapamycin^214^ were also used to downregulate necroptosis. Several agents, including Geldanamycin,^215^, ^216^, ^217^ Thio‐benzoxazepinones,^218^ Bardoxolone derivatives 20,^219^ and Chiral benzothiazole,^220^ could inhibit necroptosis by downregulating the expression of the key molecules. Phenytoin,^184^ a clinically used anticonvulsant, could inhibit RIPK1 activity in epilepsy and breast cancer. Moreover, the traditional Chinese medicine drugs could inhibit necroptosis such as aucubin,^221^ wogonin,^222^ patchouli alcohol,^223^ and cassia auriculata leaf extract.^224^ In conclusion, drugs and agents that inhibit necroptosis or the expression of RIPK1, RIPK3, and MLKL may have therapeutic potential for human disease.

Nevertheless, most of the drug research on necroptosis has been carried out through in vitro experiments or animal models. Therefore, the clinical feasibility of these compounds and drugs has yet to be evaluated in clinical trials. Up to now, 20 clinical trials on necroptosis and related molecules are recorded on Clinical Trials and Chinese Clinical Trial Registry (Table 4). Ten of these studies focus on the key molecules of necroptosis such as RIPK1, RIPK3, or MLKL. Other 10 studies explore therapeutic potential of small molecule inhibitors for human disease. Further clinical studies on necroptosis would provide ample evidence for the treatment of related diseases.

**TABLE 4:** Clinical trials related to necroptosis*

Source	Trial number	Conditions	Phase	Interventions	Target
Clinical Trials	NCT02385331	Molecular mechanism of neutrophil necroptosis and its roles in RA pathogenesis	Not applicable	Observational	Necroptosis
	NCT04229992	Calcium: magnesium balance, microbiota, and necroptosis and inflammation	Not applicable	Magnesium glycinate and placebo	Necroptosis
	NCT04169412	The mortality predictor effect of necroptosis in septic patient	Not applicable	Observational	RIPK3
	NCT04549727	The study the pathophysiology of NEC by using human enteroid biorepository	Not applicable	Observational	Necroptosis
	NCT02598648	Role and molecular mechanism of FXR and RIPK3 in the formation of ARDS in neonates	Not applicable	Observational	RIPK3
	NCT02965703	The preventing colorectal carcinoma effect of aspirin in colorectal adenoma patients	2	Aspirin and Placebo Observational (MLKL)	MLKL
	NCT03803774	The effect of birinapant and IMRT in locally recurrent HNSCC	1	Birinapant and IMRRT Observational (MLKL)	MLKL
	NCT04870125	The study of safe in ARDS with iCO and placebo	1	iCO and placebo Observational (RIPK3)	RIPK3
	NCT01105169	Investigational nutrigenetic studies for cancer prevention	Not applicable	Magnesium glycinate and placebo Observational (MLKL)	MLKL
	NCT04676711	A study of GFH312 in healthy subjects	1	GFH312 and placebo	RIPK1
	NCT02776033	The of safety, tolerability, PK, PD, and efficacy in psoriasis with GSK2982772	2	GSK2982772 and placebo	RIPK1
	NCT02903966	GSK2982772 study in subjects with ulcerative colitis	2	GSK2982772 and placebo	RIPK1
	NCT02858492	The study GSK2982772 in severe RA	2	GSK2982772 and placebo	RIPK1
	NCT03266172	The study of MR, IR, PK, and PD in patients with GSK2982772	1	GSK2982772	RIPK1
	NCT03681951	GSK3145095 alone or combination with Pembrolizumab in advanced solid tumors patients	2	GSK3145095 Pembrolizumab	RIPK1
	NCT04982991	Single ascending dose study of SAR443820 in healthy person in China and Japan	1	SAR443820	RIPK1
	NCT04781816	Proof of concept study of SAR443122 in patients with CLEan	2	SAR443122 and placebo	RIPK1
	NCT02903966	GSK2982772 study in subjects with ulcerative colitis	2	GSK2982772 and placebo	RIPK1
	NCT04469621	The safety and effect evaluation of SAR443122 on immune system in severe COVID‐19	1b	SAR443122 and placebo	RIPK1
CCTR	ChiCTR2100046488	Serum RIPK1/RIPK3 levels in patients with acute cerebral infarction	Not applicable	Observational	RIPK1‐RIPK3

ARDS, acute respiratory distress syndrome; CCTR, Chinese clinical trial registry; CLEan, Cutaneous Lupus Erythematosus; FXR, Farnesoid X Receptor; HNSCC, head and neck squamous cell carcinoma; IR, immediate release; PK, pharmacokinetics; IMRRT, intensity modulated re‐irradiation therapy; MR, modified release; NEC, necrotizing enterocolitis; PD, pharmacodynamics; RA, rheumatoid arthritis.

* All the information is from the websites of clinical trials (https://clinicaltrials.gov/) and Chinese clinical trial registry (http://www.chictr.org.cn).

## 4 CONCLUSIONS

Necroptosis, as a unique form of cell death, is mediated mainly by RIPK1/RIPK3/MLKL complex, independent of activation of caspase signaling pathway. More and more researches had found that necroptosis is widely involved in biological activities of humans and animals. Clarifying the molecular mechanism of necroptosis and its role in disease and tissue homeostasis is of great significance for harnessing its characteristics to combat disease and maintain homeostasis. The present review summarizes the laboratorial research and clinical applications of inhibitors and inducers of necroptosis pathway including small molecule inhibitors, chemicals, traditional Chinese medicine drugs, biological agents, and other drugs in various pathological conditions. Due to its important role in vital biological activities, the regulation of necroptosis and its key molecules is expected to have broad therapeutic prospects in the fields of health maintenance and disease treatment. However, one concern is that most of the drug research on necroptosis has been carried out through in vitro experiments or animal models, therefore, the clinical feasibility of these compounds and drugs has yet to be evaluated in clinical trials.

## CONFLICT OF INTEREST

The authors declare no conflict of interest.

## ETHICS APPROVAL

No ethical approval was required for this study.

## AUTHOR CONTRIBUTION

The contribution of the article confirmed as follows: ZLZ designed the study; analysis, interpretation, and critical revision for important intellectual content were performed by SZY, ZN, LLT, and DX; LXX, XX, and RYY collected the literatures and drafted manuscript. All authors approved the article for publication.

## Data Availability

Some or all data, models, or clinical trials or used during the study are available in a repository or online in accordance with funder data retention policies (Provide full citations.)
